# Evaluating regression and probabilistic methods for ECG-based electrolyte prediction

**DOI:** 10.1038/s41598-024-65223-w

**Published:** 2024-07-03

**Authors:** Philipp von Bachmann, Daniel Gedon, Fredrik K. Gustafsson, Antônio H. Ribeiro, Erik Lampa, Stefan Gustafsson, Johan Sundström, Thomas B. Schön

**Affiliations:** 1 Department of Computer Science, University of Tübingen, Tübingen, Germany; 2 Department of Information Technology, Uppsala University, Uppsala, Sweden; 3 Department of Medical Epidemiology and Biostatistics, Karolinska Institutet, Stockholm, Sweden; 4 Clinical Epidemiology Unit, Department of Medical Sciences, Uppsala University, Uppsala, Sweden; 5 George Institute for Global Health, University of New South Wales, Sydney, Australia

**Keywords:** ECGs, Electrolytes, Probabilistic deep learning, Regression, Uncertainty estimation, Cardiology, Computer science, Biomedical engineering

## Abstract

Imbalances in electrolyte concentrations can have severe consequences, but accurate and accessible measurements could improve patient outcomes. The current measurement method based on blood tests is accurate but invasive and time-consuming and is often unavailable for example in remote locations or an ambulance setting. In this paper, we explore the use of deep neural networks (DNNs) for regression tasks to accurately predict continuous electrolyte concentrations from electrocardiograms (ECGs), a quick and widely adopted tool. We analyze our DNN models on a novel dataset of over 290,000 ECGs across four major electrolytes and compare their performance with traditional machine learning models. For improved understanding, we also study the full spectrum from continuous predictions to a binary classification of extreme concentration levels. Finally, we investigate probabilistic regression approaches and explore uncertainty estimates for enhanced clinical usefulness. Our results show that DNNs outperform traditional models but model performance varies significantly across different electrolytes. While discretization leads to good classification performance, it does not address the original problem of continuous concentration level prediction. Probabilistic regression has practical potential, but our uncertainty estimates are not perfectly calibrated. Our study is therefore a first step towards developing an accurate and reliable ECG-based method for electrolyte concentration level prediction—a method with high potential impact within multiple clinical scenarios.

## Introduction

Electrolytes such as potassium or calcium influence the water and acid-base balance in the human body and ensure the proper functioning of muscles, brain and heart^1,2^. Electrolyte imbalances are frequently observed in hospitalized patients, with abnormal potassium levels affecting around 25% of them^3,4^. These imbalances can lead to severe heart conditions, such as arrhythmia and cardiac arrest^5^.

Electrolyte imbalances are challenging to detect as symptoms often do not appear until the imbalance is severe. Blood tests provide accurate measurements of electrolyte concentrations but are invasive, slow, and inaccessible from remote locations. Electrolytes have known but complex relationships with the electrocardiogram (ECG) since they directly influence heart function^6^. An ECG measures the electrical activity of the heart, it is low-cost and a widely available routine diagnostic tool for heart-related conditions in primary and specialized care. Developing an automated method to extract accurate electrolyte concentration measurements directly from the ECG could provide non-invasive, convenient, and rapid electrolyte monitoring for a large population. Such ECG-based methods would be particularly useful in rural areas relying on telehealth setups, or in an ambulance setting where blood laboratory analysis equipment typically is unavailable.

Computer-based automatic processing of ECGs is an established technology^7^. Recently, deep neural networks (DNNs) have shown promise as an alternative to traditional methods, which use hand-crafted features in combination with simple models, in the classification of cardiac diseases with known ECG patterns^8–10^. DNNs have also demonstrated success in detecting patterns that are not easily identifiable by traditional electrocardiographic analysis. For instance, models can detect myocardial infarction in ECG exams without elevation of the so-called ST segment within the ECG^11^ and predict the risk of mortality^12,13^, atrial fibrillation^14,15^, and left ventricular dysfunction^16^ directly from the ECG. Most of these models are predictive of outcomes even for seemingly normal ECGs.

DNNs have been extensively studied for ECG-based classification problems and have consistently outperformed traditional machine learning models^15,17^, prompting interest in using DNNs for automatic electrolyte prediction. However, since electrolyte concentration levels are *continuous*, the prediction is naturally formulated as a *regression* problem. While there are several regression methods using DNNs in the general literature^18–22^, few have been applied to ECG-based electrolyte prediction. The most common and simple method of DNN-based regression is known as *deep direct regression*, in which a DNN directly predicts continuous values by minimizing the mean-squared error between predicted and observed values^23^. In contrast, earlier studies on electrolyte prediction either used manually engineered ECG features combined with simple models^24,25^, focused on classifying abnormal hypo (low) and hyper (high) concentration levels^17,26^, or discretized concentration levels and applied an approach similar to ordinal regression^27^. Moreover, Lin et al.^27^ only studied the prediction of a single electrolyte (potassium).

In this work, we investigate the feasibility of utilizing DNNs to predict the continuous concentration levels of electrolytes directly from ECGs. Initially, we employ the deep direct regression approach and evaluate its regression accuracy for four major electrolytes. Our analysis reveals that the performance of the DNN model varies considerably across different electrolytes. Furthermore, we compare the performance of DNNs with that of traditional models, such as Gradient Boosting and Random Forest, and demonstrate the superior performance of DNNs in ECG-based regression. To perform this analysis, we utilize a novel large-scale dataset comprising over 290,000 ECGs collected from emergency departments in Swedish hospitals.

In cases when deep direct regression fails to accurately predict the continuous level, we study the prediction problem in more detail. We discretize the electrolyte concentration level and train classification models, with an increasing number of classes, studying the full spectrum: from continuous prediction to a binary classification of extreme concentration levels. This provides insights into the inherent difficulty of the prediction problem and enables us to extract as fine-grained predictions as possible, for different electrolytes. If the direct regression model learns a clear relationship between inputs and targets, we also extend the model to a *probabilistic regression* approach^28^, which provides uncertainty estimates for the predictions and enhances the clinical usefulness of the regression model. We evaluate these uncertainty estimates on both in-distribution and out-of-distribution data.

The paper is structured according to the following. First, in section “Methods” we explain our method starting with our dataset, provide background on regression with deep neural networks and describe our modelling and training approach. In section “Results”, we start with our results on deep direct regression, then highlight the performance using classification and ordinal regression methods and finally discuss the performance of probabilistic regression approaches. In section “Discussion”, we consider open points within the manuscript and put them into a wider context as well as discussing the limitations of our approach. In section “Conclusion” we summarize the main findings and discuss potential directions for future work.

## Methods

### Dataset

We use data from adult patients attending six emergency departments in the Stockholm area, Sweden, between 2009 and 2017. The ECG recordings are linked through unique patient identifiers to blood measurements of electrolyte concentration levels of potassium, calcium, sodium and creatinine, extracted from electronic health records with laboratory measurements. Inclusion filters are applied to only include data where the ECG and blood measurement are acquired within $\pm \,\,60~$min. Larger time frames would enable more patients in our study, but at the cost of lower label quality. Note: Potassium, calcium and sodium are electrolytes, while creatinine is a blood biomarker. In this study, however, we refer to creatinine as an electrolyte for simplicity.

Standard 10-second 12-lead ECGs are recorded, where we use the 8 independent leads since the remaining ones are mathematically redundant. Each lead provides measurements over time of the electrical activity of the heart from a different angle. The data is sampled, producing an ECG trace of size $leads \times samples$, where samples refer to the number of temporal samples. We pre-process all ECG recordings to a sampling frequency of 400 Hz and pad with zeros to obtain 4096 samples, i.e. approximately 10 s. We further apply a high-pass filter to remove biases and low-frequency trends, and finally remove possible power line noise using a notch filter. The ground truth electrolyte concentration levels are obtained by blood tests. Details on pre-processing are provided in Appendix A.3 of the Supplementary Material.

We split our datasets into training/validation and test sets. $70\,\%$ of the patients are used for model development including training/validation. The remaining $30\,\%$ are split further into two distinct test sets containing $10\,\%$ and $20\,\%$ of the complete data, respectively, to test the model in different scenarios. This implies that there are no patients with ECG records that are in both the training and test set. The first split, denoted as the *temporal test set*, contains $10\,\%$ of the data and consists of patients whose ECGs were recorded with admission date 2017-01-01 or after. Therefore, the patient ECGs are temporally separated from the ECGs of the training/validation set which were recorded with admission date before 2017-01-01. This temporal test set is used to observe changes in recordings over time and the susceptibility of the model to such changes—a highly practical scenario. For example, for our dataset, there was a restructuring of the Stockholm emergency department logistic during the data collection period, which potentially changes the composition of patients. The second split, denoted as the *random test set*, contains $20\,\%$ of the patients that were sampled at random with admission date before 2017-01-01. Therefore the patient ECGs are temporally overlapping with the training/validation set. This is the common type of test set that evaluates settings where the model is continuously updated over time. Note that these admission dates are the initial admission dates of the patients. Thus, while all patients in the temporal test set have an initial admission of 2017-01-01 or later, patients in the training/validation and random test set could have additional ECG recordings after 2017-01-01. Thus, to have complete separation from the temporal test set and avoid data leakage, we removed these ECGs. In the paper, we mainly present results on the random test set while complementing results for the temporal test set are in the Supplementary Material.

We obtain four datasets—one for each electrolyte. The number of patients ranges between $79,577$ and $166,908$, with between $126,970$ and $295,606$ ECGs in total. In Table 1 we provide summary statistics of the datasets with detailed information in Appendix A.2 and Fig. S-1. For a given patient, multiple blood measurements and multiple ECGs are sometimes recorded within the selected $\pm 60$ minute time frame. We select the median electrolyte value and assign it to all ECGs for training. We consider multiple ECGs during training as a form of data augmentation. In the validation and test sets, we use only the first ECG. Details and comparisons with datasets from literature are in Appendix A.2.

The study was approved by the Swedish Ethical Review Authority, application number 2020–01654. Informed consent was waived in this study by all applicable ethics review boards, i.e. the Region Stockholm Ethical Review Board and the Swedish Ethical Review Authority. All methods were performed in accordance with the relevant guidelines and regulations.

**Table 1:** Characteristics of our datasets.

		Potassium	Calcium	Sodium	Creatinine
Patients		165,508	79,577	163,610	166,908
ECG recordings		290,889	125,970	288,891	295,606
% Male		49.38	48.71	49.07	49.22
Age	mean	61.26	60.47	61.41	61.34
	sd	19.61	20.03	19.69	19.61
Minutes diff (abs)	mean	16.28	12.68	15.92	16.24
	sd	15.04	14.05	14.91	15.01
Concentration	mean	3.99	2.29	138.93	90.55
	sd	0.50	0.13	3.82	71.00

### Background: regression approaches with deep neural networks

**Figure 1:**
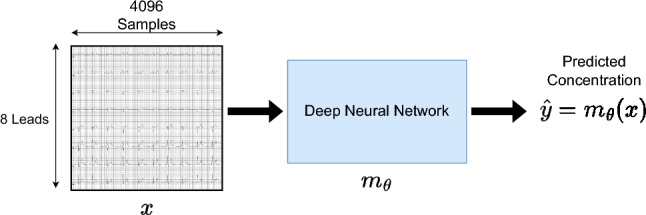
Model overview: we employ the *deep direct regression* approach, training a DNN $m_\theta$ to directly output predicted electrolyte concentration levels $\hat{y} = m_\theta (x)$ for given inputs *x*. The inputs *x* are ECG traces of size $leads \times samples$.

The goal in a regression problem is to predict a continuous target $y\in \mathbb {R}$ for an input *x*, given a training dataset $\mathcal {D} = \{x_i, y_i\}_{i=1}^n$ of *n* data points. This is achieved by training a model $m_\theta$ with parameters $\theta$ such that a loss function is minimized on $\mathcal {D}$. In the common *deep direct regression* approach, the model is a DNN outputting continuous predictions, $\hat{y} = m_\theta (x)$, using the Mean-Squared Error (MSE) as loss function. In our specific problem of ECG-based electrolyte prediction, the input *x* is an ECG trace of size $leads \times samples$, and $\hat{y} = m_\theta (x)$ is the predicted electrolyte concentration level. Figure 1 gives an overview of this deep direct regression approach.

**Probabilistic regression** The predictions $\hat{y} = m_\theta (x)$ output by deep direct regression models do however not capture any measure of uncertainty. In medical applications, where predictions might impact the treatment of patients, this lack of uncertainty is especially problematic. Probabilistic regression aims to solve this problem by estimating different types of uncertainties^28–30^. (1) *Aleatoric uncertainty* captures irreducible ambiguity from the experiment itself. One example is noise from a measurement device. (2) *Epistemic uncertainty* refers to a lack of knowledge and is therefore reducible. Out-of-distribution (OOD) data is one example where epistemic uncertainty is expected to be high. Aleatoric uncertainty can be estimated by explicitly modeling the conditional distribution *p*(*y*|*x*). Assuming a Gaussian likelihood leads to the parametric model $p(y \vert x; \theta )=\mathcal {N}\big ( y; \mu _\theta (x) , \sigma ^2_\theta (x)\big )$, where the DNN $m_\theta$ outputs both the mean $\mu$ and variance $\sigma ^2$, i.e.  $m_\theta (x) = [{\mu _{\theta }(x) \quad \sigma ^2_{\theta }(x)}]^{\textsf{T}}$. The mean is used as a target prediction, $\hat{y} = \mu _\theta (x)$, whereas the variance $\sigma ^2_{\theta }(x)$ is interpreted as an estimate of input-dependent aleatoric uncertainty. The DNN is trained by minimizing the corresponding negative log-likelihood $- \sum _{i=1}^{n} \log p(y_i | x_i; \theta )$.

This approach does however not capture epistemic uncertainty. One way to add this uncertainty is by treating the model parameters $\theta$ according to the Bayesian framework^31^ and learning a *posterior* probability distribution over the parameters. Ensemble methods^30,32^ constitute a simple approach to estimating epistemic uncertainty. Multiple models are trained and the uncertainty is estimated by the variance of the prediction over all models. Ensemble methods usually improve the regression accuracy of the model and have been shown to be highly competitive baselines for uncertainty estimation methods^33,34^. Another common method is the Laplace approximation^35^, which approximates the *posterior* distribution $p(\theta \vert \mathcal {D})$ with a Gaussian, $p(\theta \vert \mathcal {D}) \approx \mathcal {N}(\theta ; \theta _{\text {MAP}}, \Sigma )$, where the inverse of the covariance matrix $\Sigma ^{-1}=-\nabla _{\theta }^2 \log p(\mathcal {D}, \theta ) \vert _{\theta _\text {MAP}}$ is the negative Hessian matrix, evaluated at the *maximum-a-posteriori* estimate $\theta _{\text {MAP}}$. Laplace approximations can be applied post-hoc to a pre-trained model with reduced computational complexity^36,37^.

**Simplifying regression via discretization** If the accurate prediction of the continuous target $y\in \mathbb {R}$ is unachievable or not required, a regression problem can be simplified into a standard classification problem by discretizing the targets. Specifically, the target range is divided into *k* intervals and each target *y* is assigned to the respective interval^38^. The model now outputs a distribution over *k* classes and predictions are made by the class with maximum probability.

Usually, the Cross-Entropy (CE) loss is used to train the model, which can however lead to rank inconsistency of the original continuous problem. This implies that for a predicted class $\tilde{y}$ corresponding to a certain target interval, the probability for the classes corresponding to neighbouring intervals do not necessarily decrease monotonically away from the predicted class. To address this issue, Cao et al.^39^ proposed rank-consistency *ordinal regression*. The output of the model is changed to denote the probability that the target *y* is larger than or equal to the lower bound of the corresponding interval of class *j*. The model is trained with binary CE loss, and class predictions are computed as $\tilde{y} = 1 + \sum _{j=1}^{k} p_\theta (x)_j$, where $p_\theta (x)_j = \mathbbm {1}_{m_\theta (x)_j > 0.5}$.

### Models and training procedures

**Baseline comparison** We first conduct a baseline performance comparison of our DNN model with different machine learning models. The raw ECG trace is of size $leads \times samples$ (i.e., $8 \times 4096$), yielding $32,768$ features. For instance for potassium, we have $290,889$ ECG traces in our dataset. In contrast to training deep models, most traditional machine learning models use the full dataset in every iteration of training. However, given our dataset, this would be computationally infeasible. Therefore, we conduct two types of baseline comparisons. First, we *reduce the feature dimension* to ensure computational feasibility. The reduced data are applied to traditional machine learning models, namely linear regression, Gradient Boosting^40^, and Random Forest^41^. To reduce the feature dimension, we employ principal component analysis (PCA) as a standard pre-processing step for the raw ECG traces. While PCA-based pre-processing is established for ECGs, it is typically applied to individual ECGs separately^42^. Given our large dataset, this approach would however be very computationally expensive. Thus we instead perform PCA on the entire dataset by vectorizing the $8\times 4096$ features and then reducing them. The reduced dimension is set to 256, based on the eigenvalue distribution shown in Fig. S-3, which explains approximately 60 % of the variance. However, this dimensionality reduction might still remove important information from the raw data. Hence, we perform a second baseline comparison, where we keep all features but ensure computational feasibility by *training in a batch-wise manner*—the usual training strategy for deep networks. In this scenario, we compare with batch-wise linear regression as well as a small 3-layer multi-layer perceptron (MLP) on the raw ECG input. The batch-wise training is done equally to our DNN.

**Model architecture** For the choice of DNN architecture, reviews note that convolutional models such as Residual Networks (ResNets) are the dominant deep architecture for ECG-based prediction modelling^43,44^. The authors in^10^ also experimented with vectogram linear transformation for dimensionality reduction, Long Short-Term Memory networks (LSTMs) and a Visual Geometry Group (VGG) convolutional architecture, but used a ResNet. Hence, we use the ResNet backbone network from^10,13^ as a feature extractor in all DNN models. For details on the model, see Appendix B. Our methodological approach is however model-agnostic and any architecture with high performance could be utilized instead.

**Model training** For *deep direct regression*, the DNN $m_\theta$ consists of the ResNet backbone that extracts a feature vector for each given input *x* and a small network head that takes this feature vector as input and produces a predicted target, $\hat{y} = m_\theta (x)$. The DNN is trained from scratch using the MSE loss. During training, we apply z-score normalization of the targets *y* to obtain a similar target distribution across all electrolytes. We then discretize the targets into *k* intervals, and train both classification and ordinal regression models, as described in “Simplifying regression via discretization” above. The network head of the direct regression DNN is modified to output *k* values instead. For ordinal regression, we train using binary CE loss and for classification using the CE loss. All models are trained for 30 epochs and the final model is selected from the best validation loss. If not specified otherwise, we train each model with 5 different seeds and report the mean and standard deviation (sd) for all results.

For *probabilistic regression*, we create a Gaussian model $\mathcal {N}\big ( y; \mu _\theta (x), \sigma ^2_\theta (x)\big )$ by extending the direct regression DNN with a second network head that outputs the variance $\sigma ^2_\theta (x)$. The model is trained by minimizing the corresponding negative log-likelihood. We train an ensemble of 5 Gaussian models, and then extract three different uncertainty estimates: (1) *Aleatoric* uncertainty is given by the average predicted variance $\sigma ^2_\theta (x)$ (denoted aleatoric Gaussian). (2) *Epistemic uncertainty* is computed as the variance of the predicted mean $\mu _\theta (x)$ over the 5 ensemble members (denoted epistemic ensemble). (3) We additionally define an *epistemic uncertainty* by fitting a Laplace approximation after training using the Laplace library^37^ (denoted epistemic Laplace). The approximation is fit to the last layer of the mean network head by approximating the full Hessian. We report the average epistemic Laplace uncertainty over the 5 ensemble members.

The code is implemented in PyTorch^45^ and models are trained on a single Nvidia A100 GPU. Further training details are in Appendix B. Our complete implementation code and the trained models are publicly available at https://github.com/philippvb/ecg-electrolyte-regression.

## Results

In this section, we start by discussing the results obtained through deep direct regression. Following that, we compare classification and ordinal regression in the context of discretized regression. Finally, we focus on potassium for probabilistic regression. In summary, our results show that: (1) DNNs outperform traditional machine learning models in the ECG-based electrolyte regression task. (2) Model performance varies significantly across different electrolytes. (3) While discretization leads to good classification performance, it does not address the original problem of continuous concentration level prediction. (4) Probabilistic regression has practical potential, but the uncertainty estimates are not perfectly calibrated.

**Table 2:** Regression comparison with baseline models on the random test set for potassium.

	Model	MSE	MAE
Feature reduction with PCA to 256 dims.	Gradient Boosting	0.215	0.340
	Random Forest	0.220	0.346
	Linear Regression	0.220	0.344
Batch-wise training	Linear Regression	0.215	0.338
	3-layer MLP	0.218	0.346
	ResNet	0.152	0.285

### Deep direct regression

**Baseline comparison** We validate our ResNet architecture with baseline comparisons, using PCA-based feature reduction or batch-wise training to enable computational feasibility. The results obtained for potassium on the random test set are presented in Table 2. It is worth noting that the ground truth regression targets for potassium have a variance of 0.220. Simply predicting the mean target value for all inputs (resulting in an MSE equal to the variance) would thus achieve similar performance to all baseline models, indicating that the baselines fail to capture a clear relationship between ECG and potassium concentration. This behaviour is independent of the method used to ensure computational feasibility. In contrast, our DNN model achieves a significantly lower MSE and mean absolute error (MAE), implying that it has learned the true underlying relationship effectively. DNNs thus clearly outperform traditional machine learning models on this regression task.

**Figure 2:**
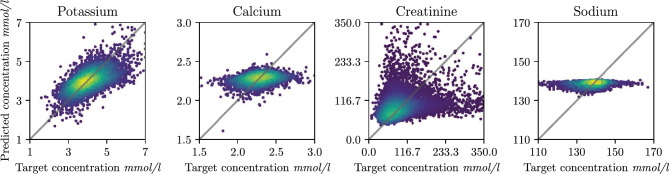
Regression scatter plot: results of our deep direct regression model on the random test set, for all four electrolytes. The diagonal depicts the optimal fit and the point density is indicated by colour (yellow: high, blue: low) from a Gaussian kernel density estimation (KDE).

**Table 3:** Main regression results: results of our deep direct regression model on both the random and temporal test set, for all four electrolytes.

	Random test set				Temporal test set		
	MSE (sd)	MAE (sd)	*R* (sd)	$\rho$ (sd)	MAE (sd)	*R* (sd)	$\rho$ (sd)
Potassium	0.152 (0.026)	0.285 (0.015)	0.582 (0.043)	0.561 (0.021)	0.262 (0.013)	0.604 (0.034)	0.578 (0.023)
Calcium	0.015 ($2\textrm{e}{-4}$)	0.088 ($5\textrm{e}{-4}$)	0.272 (0.013)	0.252 (0.010)	0.106 ($3\textrm{e}{-4}$)	0.257 (0.009)	0.240 (0.009)
Sodium	12.59 (0.111)	2.512 (0.016)	0.219 (0.015)	0.191 (0.013)	2.390 (0.009)	0.265 (0.018)	0.227 (0.011)
Creatinine	3719 (86.04)	26.69 (1.118)	0.353 (0.059)	0.422 (0.045)	24.50 (1.298)	0.366 (0.047)	0.367 (0.035)

Targets are not normalized. The Pearson correlation coefficient is denoted by *R*, Spearman correlation by $\rho$.

**Main results**

Figure 2 depicts the results of our deep direct regression model for potassium, calcium, creatinine and sodium, plotting predictions $\hat{y}$ against targets *y*. For potassium, the data points concentrate along the diagonal, indicating an overall good fit. For calcium and sodium, the predictions are horizontally aligned, meaning that the model mainly predicts the mean target value of the train dataset for all inputs *x*.

Creatinine shows an overall positive trend, but the model seems to suffer from the high variance for higher target values, making predictions for these values noisy. Since the main behavior is captured by potassium and calcium, we will primarily focus on them in the main text.

Quantitative results on both the random and temporal test set are presented in Table 3. There, the MSE and MAE do not directly reflect the performance difference between calcium and potassium observed in Fig. 2, as calcium shows significantly lower errors. To understand these opposing results, we investigate the dataset distributions. The variance in the ground truth electrolyte levels is significantly lower for calcium with 0.016 compared to potassium with 0.220. Thus, predicting the mean training target value for calcium will result in a lower MSE without learning the relationship between input and target. Computing errors with normalized targets give MSE values which better reflect the performance difference, as TableS-2 shows.

In terms of correlation (Pearson and Spearman) between true and predicted values, we can see in Table 3 that again potassium reaches the highest values, followed by creatinine. In contrast, calcium and sodium reach correlation values that do not indicate a good fit.

From the results in Table 3, we do not observe a significant drop in performance when evaluating our model on the temporal test set. In fact, the MAE is often slightly lower than for the random test set and the correlation slightly higher. This is the first indication that our model is agnostic to real-world data collection changes and distribution shifts. Other works that report regression performance of potassium concentrations obtain MAEs of 0.53^27^ and 0.50^46^, compared to which our model obtains superior performance.

**Figure 3:**
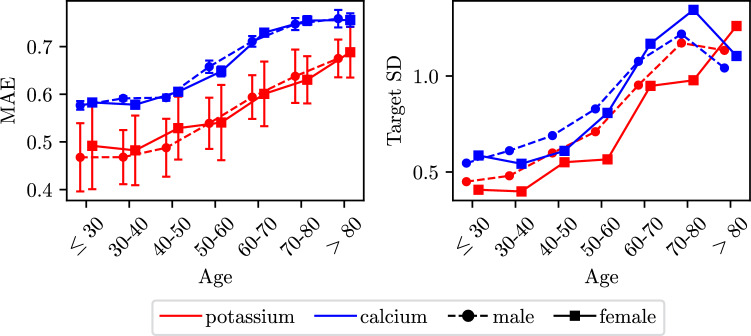
Stratified regression results: Left: MAE of regression task stratified for different electrolytes by age and sex. Targets are normalized to obtain MAE values which are comparable across the different electrolytes. Right: Corresponding standard deviations of the target values stratified in the same way.

**Stratification** To further analyze our results, we stratify them according to the age and sex of the patients (note that we do not have access to any other demographic factors in the datasets) in Fig. 3 (left). We observe that our results are independent of sex, but that the MAE has a positive correlation with patient age. Comparing with Fig. 3 (right) we see that this correlation is largely expected since the variance of the ground truth target values also increases with age. Correctly predicting the electrolyte concentration levels is thus inherently more difficult for older patients.

### Classification and ordinal regression

We compare classification and ordinal regression in the simplified setting with discretized targets *y*. We consider increasingly fine-grained predictions by varying the number of intervals *k*. The class intervals are defined for each electrolyte separately: For *k* = 3 classes, we define the lower and upper interval bounds by $\mu \pm 2 \sigma$. For *k* > 3 classes we add evenly spaced interval bounds in between the extreme bounds. For binary classification (*k* = 2) we consider the hypo/hyper definitions from Kwon et al.^17^ (Calcium: 2.0/2.75; potassium: 3.5/5.5; creatinine: 3.5/5.3; sodium: 130/150. Values in mmol/l. For creatinine, we default to $\mu \pm 2\sigma$ as Kwon et al.^17^ do not consider creatinine). For evaluation, we compute Receiver-Operating-Characteristic (ROC) curves for the cumulative classification $p(\tilde{y} \le i),\,i=1,\dots ,k$, leading to $k-1$ individual curves.

**Figure 4:**
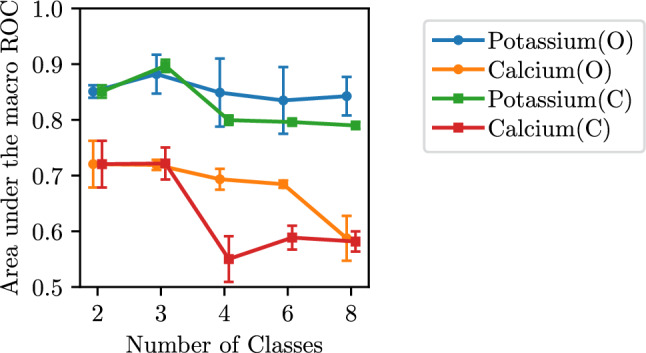
Macro ROC for varying number of classes: O: Ordinal regression; C: classification models. For 2 classes we average the hypo and hyper results (see Table 4).

**Table 4:** Binary classification AUROC.

	*n* Data	Hypo	Hyper
**Potassium**		*(Bounds 3.5 / 5.5 mmol/l)*	
Ours	290 889	0.809 (0.003)	0.892 (0.009)
Lin et al.^27^	66 321	0.926	0.958
Galloway et al.^26^	2 835 059	N/A	0.865
Kwon et al.^17^	83 449	0.866	0.945
**Calcium**		*(Bounds 2.0 / 2.75 mmol/l)*	
Ours	125 970	0.779 (0.012)	0.660 (0.036)
Kwon et al.^17^	83 449	0.901	0.905

Figure 4 shows the area under the macro averaged ROC (AUmROC) for different number of classes *k*. The AUmROC simply averages the obtained $k-1$ AUROC values. For all *k*, the prediction performance on calcium is worse than on potassium. When increasing the number of classes, we observe a drop in AUmROC which implies that fine-grained predictions are increasingly difficult. This effect is also stronger for calcium. Together with Fig. 2, these results clearly suggest that accurate prediction of concentration levels is inherently more difficult for calcium than for potassium. Comparing classification against ordinal regression in Fig. 4, the latter decreases less in AUmROC for more classes. In this discretized regression setting, ordinal regression can thus improve performance compared to standard classification models.

We now convert the class predictions into electrolyte concentration levels by mapping to the mean of the predicted class interval and computing the error to the continuous targets. The results in Fig. S-4show that the MAE decreases with more classes for potassium but stays mostly constant for calcium. However, the MAE is never lower than the corresponding direct regression MAE from Table 3. While discretization thus can lead to good *classification* performance, it does not help solve the original problem of predicting continuous concentration levels.

For binary classification, we compare with results from the literature in Table 4. The comparisons are not entirely fair since data collection and dataset size is different between all works. For potassium, our model reaches a slightly lower AUROC for both imbalances than 2 out of 3 works from the literature. For calcium, Kwon et al.^17^ reach a significantly higher AUROC, indicating that specialized models might outperform our approach which relies on a standard ResNet.

### Probabilistic regression

**Figure 5:**
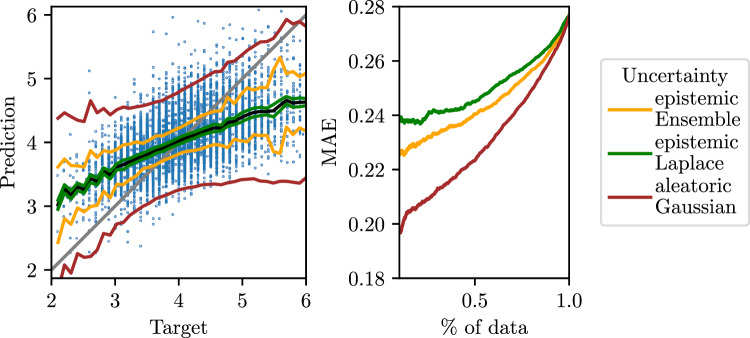
Regression uncertainty for potassium: Left: Prediction vs target plot as in Fig. 2. The black line indicates mean prediction $\mu$. The coloured lines show $\mu \pm 2 \sigma$ for different types of uncertainties. Right: Sparsification plot.

Here, we focus on potassium as the only electrolyte for which direct regression learns a clear relationship between inputs and targets. Figure 5 (left) shows the three uncertainty estimates defined in “Methods”, for different target levels. Aleatoric Gaussian uncertainty fits the noise in the predictions quite well, since it increases towards the extremes, where predictions become noisy and the error increases. In comparison, both epistemic variances are smaller, which is expected due to the large size of the underlying training dataset. Epistemic Laplace is the smallest and almost constant. Epistemic from the ensemble increases towards the extreme values, similar to the aleatoric uncertainty.

Meaningful uncertainty estimates should correlate with the error—predictions with high error should also have high uncertainty. The sparsification plot in Fig. 5 (right) shows that removing the most uncertain points from the test set lowers the MAE monotonically, as desired. This effect is strongest for aleatoric Gaussian uncertainty. However, the calibration plot in Figure S-6 shows that the uncertainties are not particularly well-calibrated. Aleatoric Gaussian uncertainty has the highest correlation with the MSE, see Table S-5. The correlation can be increased by adding epistemic ensemble uncertainty.

To further measure uncertainty, we perform OOD experiments similar to Xia et al.^47^. We first add Gaussian noise to the ECG traces, controlling the strength with the signal-to-noise ratio (SNR). In Table 5 we observe that with increasing noise both the MAE and all uncertainty measures rise. This indicates that each uncertainty by itself is a useful indicator for this kind of OOD data. As a second experiment, we randomly mask a proportion of each ECG trace. The results for this experiment are provided in Table S-6 and indicate that this type of OOD data is not detected by our uncertainty quantification.

**Table 5:** Results for the OOD experiment.

	Baseline	SNR = 10	SNR = 1
MAE	0.304 (0.021)	0.330 (0.016)	0.368 (0.026)
Aleatoric Gaussian	0.389 (0.012)	0.399 (0.012)	0.480 (0.078)
Epistemic Ensemble	0.121 (0.048)	0.149 (0.041)	0.184 (0.075)
Epistemic Laplace	0.022 (0.003)	0.028 (0.009)	0.049 (0.031)

## Discussion

**Clinical relevance** In an in-hospital scenario, blood measurement with laboratory analysis is the gold standard method for accurately and reliably determining the electrolyte concentration levels of patients. However, there are multiple scenarios where ECG-based prediction models are desired.

First, in the ambulance setting, there is typically no access to onboard blood laboratory analysis equipment but it is possible to acquire ECGs. Many countries apply a telehealth setup in this scenario and send the ECGs to a coronary care unit for reading and decision-making. If the patient in the ambulance has presented with arrhythmia, which is potentially lethal, it is important to quickly identify its cause. If electrolyte disturbances could be estimated, and identified as the cause of the arrhythmia, then life-saving treatment could be started directly in the ambulance. For example, an insulin-glucose infusion, an intravenous calcium injection, or an inhalation of a beta-2-agonist are all treatments for hyperkalemia (high potassium) that could be administered. Onboard automated ECG analysis would be highly useful in this scenario.

Second, in rural areas without specialists, a remote setting with telehealth care centres is built up. One such example is the Telehealth Network of Minas Gerais, Brazil^48^ which receives up to 5 000 ECGs per day. In many locations, obtaining an ECG may be easier than obtaining a blood sample for electrolyte analysis. ECG-based models could provide crucial care for these patients which would otherwise not be possible at all.

Third, in an in-hospital setting, an ECG-based electrolyte prediction could be useful for monitoring the treatment of hyperkalemia or hypernatremia (high sodium). For hyperkalemia, monitoring that potassium is decreased fast enough is an important objective; for hypernatremia, monitoring that sodium is decreased slowly enough (to prevent potentially lethal brain edema due to osmosis) is crucial. A real-time ECG-based prediction could replace frequent blood draws in these scenarios.

Extending the prediction model with uncertainty estimation increases its clinical usefulness. (1) *Aleatoric uncertainty* captures inherent ambiguity in the data itself. For example, due to measurement noise, it is inherently more difficult to determine the electrolyte levels for some ECGs than for others. Hence, an accurate prediction of concentration level might not be possible for some ECGs. A model that properly captures aleatoric uncertainty could automatically detect such cases, enabling doctors to take appropriate action such as acquiring a new ECG or asking for blood test analysis instead. If a model captures (2) *epistemic uncertainty*, it could detect cases when the ECG being analysed during clinical deployment is out-of-distribution compared to the training data. Failing to detect such cases could lead to highly incorrect model predictions, with potentially catastrophic consequences, since the accuracy of DNN models can drop significantly on out-of-distribution examples^49,50^.

**Model performance for practical utility** From the results in Fig. 2 and Table 3, it is clear that the deep direct regression model reaches significantly better performance for potassium than the three other electrolytes. With Pearson correlations of 0.27, 0.22 and 0.35 (on the random test set), the current model is unlikely to have much practical utility for the prediction of calcium, sodium or creatinine. For potassium, the model achieved Pearson correlations of 0.58 and 0.60 on the random and temporal test sets, respectively. While significantly better than for the other electrolytes, this is still not sufficient for the model to replace the gold standard method of blood measurement with laboratory analysis. However, in the telehealth scenarios discussed above where this gold standard method is unavailable, and the alternative thus is no electrolyte assessment at all, our ECG-based model for potassium prediction has clear potential for real-world clinical utility. In particular, the model has the potential to be utilized as a pre-screening tool, identifying patients with suspected potassium imbalances who therefore should be selected for further analysis via blood testing. The level of model performance required for such real-world deployment does however depend on the specific application, and it is difficult to determine exactly to what extent our current potassium model meets those requirements. Extensive further validation would therefore need to be conducted in future work.

**Challenges in predicting calcium levels** The varying performance of our models across electrolytes requires further discussion. The difficulty in predicting calcium levels in contrast to potassium levels can be explained with their manifestation in the ECG. There is a known relationship between both electrolyte levels and a change of the ECG, which for calcium is revealed mainly in a change of the QT interval^51,52^. The range of values for calcium is however very narrow since extreme values can be lethal and it thus is tightly regulated by many mechanisms in the human body. In our dataset, about $95\,\%$ of the values are in the range $2.29\pm 0.26$, see Table 1. The electrophysiological manifestation of this change in concentration level could be negligible, as Pilia et al.^53^, Figure 2 indicates. Further, the calcium dataset size is less than half that of potassium, thus the number of patients with extreme calcium values could be insufficient to predict those reliably.

**Interlinked effects of electrolytes** The electrophysiological effects of potassium, calcium, sodium and creatinine are closely interlinked. For example, extracellular hypo- and hyperkalemia (potassium) levels promote cardiac arrhythmias, partly because of direct potassium effects, and partly because the intracellular balances of potassium, sodium and calcium are linked. Thus, hypo- and hyperkalemia directly impact sodium and calcium balances. Finally, creatinine levels can signify renal disease that can lead to hyperkalemia. This complex relationship between the studied electrolytes, together with the significantly better regression results achieved for potassium, could indicate that the summed electrophysiological effects are most tightly linked to potassium concentration. However, due to the known connection of calcium to the ECG, it is unclear if the combined electrophysiological effects could also be linked to calcium if we had a similar dataset size as for potassium.

**Relation to prior work** Most previous work on ECG-based electrolyte prediction relies on hand-crafted ECG features. These are specific characteristics such as the time between two waves, and the amplitude or slope of a wave. Frohnert et al.^24^ were the first to manually develop a relation between such features and electrolyte concentrations. More recently,^25,46,54^ rely on hand-crafted features but automatically fit the model parameters to data. Their performance is however limited. DNNs offer a different approach by jointly learning features and predictions. Convolutional neural networks (CNNs) have shown promising results for classifying different ECG patterns^8–10^. For electrolyte prediction, Galloway et al.^26^ used an 11-layer CNN to classify hyperkalemia. Lin et al.^27^ were the first to develop a DNN for regression on potassium, using an approach similar to ordinal regression by discretizing the model outputs. Hence, despite recent work on ECG-based predictions for electrolytes, it remains unclear if the common deep direct regression approach can be applied to accurately predict electrolyte concentration levels from ECGs.

Little work has gone into deep prediction models for electrolytes other than potassium. Kwon et al.^17^ studied potassium, calcium and sodium, but they only considered the simplified problem of classifying hypo and hyper conditions. We are the first to study these electrolytes in the original problem setting of regressing continuous concentration levels. Moreover, we also consider the prediction of creatinine. We further apply probabilistic regression methods for uncertainty estimation. Closest to this probabilistic setting is^47^, which proposed dataset shifts and compared the change of uncertainty for different models but concentrated exclusively on classification problems.

**Limitations** The primary focus of our work is to develop a methodology for performing regression in a medically realistic setup, rather than improving the model itself. Despite our model outperforming existing approaches, we observed that our model did not perform as well as some reference work for binary extreme value classification. Nevertheless, it is challenging to compare our results with those of other works due to differences in problem definition, such as the time interval between ECG and blood measurement. Therefore, we cannot definitively conclude that our model underperforms in this specific setting. Moreover, we should note that our model is based on certain assumptions, such as assigning ground-truth electrolyte concentration values to ECGs, which may introduce noise into our dataset.

Real-world deployment of machine learning models for electrolyte concentration level prediction, or other safety-critical medical applications, puts high requirements in terms of model accuracy and reliability. If models fail to detect electrolyte imbalances and instead incorrectly predict normal concentration levels for such cases, this could have severe consequences. In principle, these concerns can be addressed by utilizing methods that estimate the uncertainty in the model predictions. These uncertainty estimates must however also be calibrated. Otherwise, if models occasionally output highly confident yet incorrect predictions, performing uncertainty estimation risks instilling a false sense of model trustworthiness. Further work therefore still lies in improving the calibration of our uncertainty quantification, for which we present a first step in the setting of ECG-based regression.

While the performance on the temporal test set in Table 3 is an encouraging first indication of model generalizability, real-world deployment would also require a more comprehensive external validation to first be conducted. Specifically, our models were trained on a large-scale yet geographically concentrated dataset (patients attending emergency departments in the Stockholm area, Sweden) and should therefore be evaluated on external, geographically shifted patient cohorts.

## Conclusion

We trained deep models for direct regression of continuous electrolyte concentration levels from ECGs, utilizing a novel large-scale dataset comprising over 290,000 ECGs. While the model for potassium performed quite well, it struggled with the three other electrolytes. Simplifying the problem to binary classification, of clinically critical low or high levels, indicated that also those electrolytes for which the direct regression model struggled can achieve good classification performance. Defining more classes, for increasingly fine-grained predictions, we observed a sharp performance drop for electrolytes other than potassium. Our results thus strongly suggest that accurate ECG-based prediction of concentration levels is inherently more difficult for some electrolytes than for others. Retrospectively, we can justify why in our context potassium levels can be predicted better than e.g. calcium levels—yielding insights into the manifestation of electrolytes on the ECG. Future work should study this problem for an even larger set of electrolytes, in order to potentially identify other electrolytes which can be accurately predicted, and explore the possibility of using a single combined model for all electrolytes due to their medial interconnection. Comparing how different model architectures, including recurrent and transformer-based models, affect prediction accuracy is another possible direction for future work.

Given the presented prediction accuracy, the current deep direct regression model is unlikely to have much practical utility for the prediction of electrolyte concentrations of calcium, sodium or creatinine. While the model performance for potassium is not sufficient to replace the gold standard method of blood test analysis, our ECG-based model for potassium prediction still has clear potential for real-world clinical utility within different telehealth scenarios. In particular, the model has the potential to be utilized as a pre-screening tool. For real-world deployment to be possible, extensive validation does however need to be conducted in future work, determining precisely to what extent the model performance meets the requirements in different specific applications.

Consistent with prior work on ECG-based *classification* tasks, our results demonstrated that DNNs outperform traditional machine learning models, also in our *regression* setting. Therefore, while DNN models generally are more complex and less interpretable, their superior performance is a strong argument for their use also within medical domains. Moreover, by simplifying the original problem via discretization of the electrolyte concentration levels, we found that good classification accuracy can be achieved even in cases when regression models struggle. By progressively increasing the number of discretized classes, we also demonstrated how to extract as fine-grained predictions as possible.

Finally, we extended our deep direct regression model to the probabilistic regression approach and carefully analyzed the resulting uncertainty estimates. While especially the aleatoric uncertainty demonstrated potential practical usefulness e.g. via sparsification, the uncertainty estimates are not particularly well-calibrated. To achieve the ultimate goal of reliable and clinically useful prediction of electrolyte concentration levels, future work investigating possible approaches for improved uncertainty calibration is thus required.

Our study is a first step towards developing accurate and reliable ECG-based methods for continuous electrolyte concentration level prediction—methods with high potential impact within multiple clinical scenarios.

### Supplementary Information

Supplementary Information.

## Data Availability

The data that support the findings of this study are available from the Swedish Board of Health and Welfare and the included healthcare regions, but restrictions apply to the availability of these data, which were used under license for the current study, and so are not publicly available. Data are however available from the corresponding author upon reasonable request and with permission of the Swedish Board of Health and Welfare and the included healthcare regions. Code is freely available at https://github.com/philippvb/ecg-electrolyte-regression and pre-trained models are available at https://zenodo.org/records/7456316#.Y6CHPS8w1qs.
