# Supplementary material for: Evaluating regression and probabilistic methods for ECG-based electrolyte prediction
Source: Sci Rep. 2024 Jul 3;14:15273. doi: 10.1038/s41598-024-65223-w (PMC11222546; doi:10.1038/s41598-024-65223-w)
Supplement: Supplementary file 1 — Supplementary Information. [file 41598_2024_65223_MOESM1_ESM.pdf]

Supplementary Material

A Dataset

A.1 Clarification of Electrolyte Definitions

We note that potassium, calcium and sodium are by definition electrolytes but creatinine is an abundant blood biomarker. For ease of reading descriptions, we denote creatinine as an electrolyte in this study. The reason to include creatinine is the availability of large amounts of data and the general medical interest in its predictions. In some figures and tables in this appendix, creatinine is denoted "pcreatinine".

A.2 Dataset Characteristics

The characteristics of our four datasets are given in Table 1. More generally a population of emergency room patients with electrolyte imbalances has characteristics as described in Balcı et al. [55]. We include data from all-comer patients to the emergency room with ≥ 18 years old with the only restriction that there is a blood biomarker test and ECG collected within 60 minutes. We have a varying number of patients in each dataset because not all electrolyte concentration values are available for all patients. Note that we use an inclusion filter of ± 60 minutes between ECG and blood measurement. We can compare our datasets with related work from the literature:

- Lin et al. [27] use 66 321 ECG recordings from 40 180 patients and related potassium concentration in a time frame of ± 60 minutes.
- Galloway et al. [26] use 2 835 059 ECG recordings from 787 661 patients and related potassium concentrations. The authors develop their model on $60\,\% (= 449\,380)$ of the patients. All ECGs were recorded within 4 hours before potassium measurements.
- Kwon et al. [17] have 92 140 patients, whereof 48 356 patients were used for model development with 83 449 ECGs. The study considered potassium, sodium and calcium within ± 30 minutes of ECG recordings.

We analysed our datasets in more detail to observe possible causes of errors or shortcuts for our model. In Figure S-1 we show histograms of age, recording year and the time difference between ECG recording and blood measurement. In Figure S-2 we show the distribution of electrolyte concentrations for all four electrolytes, which shows a Normal distribution for all electrolytes except for creatinine which is skewed towards large values. In order to validate our inclusion filter of ± 60 minutes, we analyze the concentration of electrolytes vs the time difference and observe no clear change of concentration value over time. A similar analysis is done for age and sex. Here, we observe that older patients tend to have more extreme electrolyte concentration values for all four electrolytes.

A.3 Pre-processing

For the high-pass filter to remove the baseline (trends and low frequencies), we use an elliptic filter with a cut-off frequency of 0.8 Hz and an attenuation of 40 dB which is applied to the forward and reverse direction to avoid phase distortions. We additionally include a notch filter after observing that some ECGs are distorted by power line noise. The notch filter removes the 50 Hz with a quality factor of 30. Also, this filter is applied to the forward and reverse directions for the same reason. We use the pre-processing from the public library `github.com/antonior92/ecg-preprocessing`.

For the traditional machine learning methods, which we compare in "Results", "Deep Direct Regression", we further apply Principal Components Analysis (PCA) to reduce the dimensionality of the data. Here, we first concatenate all leads to get a 1D signal of length $leads \cdot samples = 8 \cdot 4096 = 32\,768$. Then we fit PCA on our train dataset. We choose the number of principal components based on the eigenvalues in Figure S-3. We see that the eigenvalues decrease fast and start to converge between 200 and 300, which is why we choose to use 256 components.

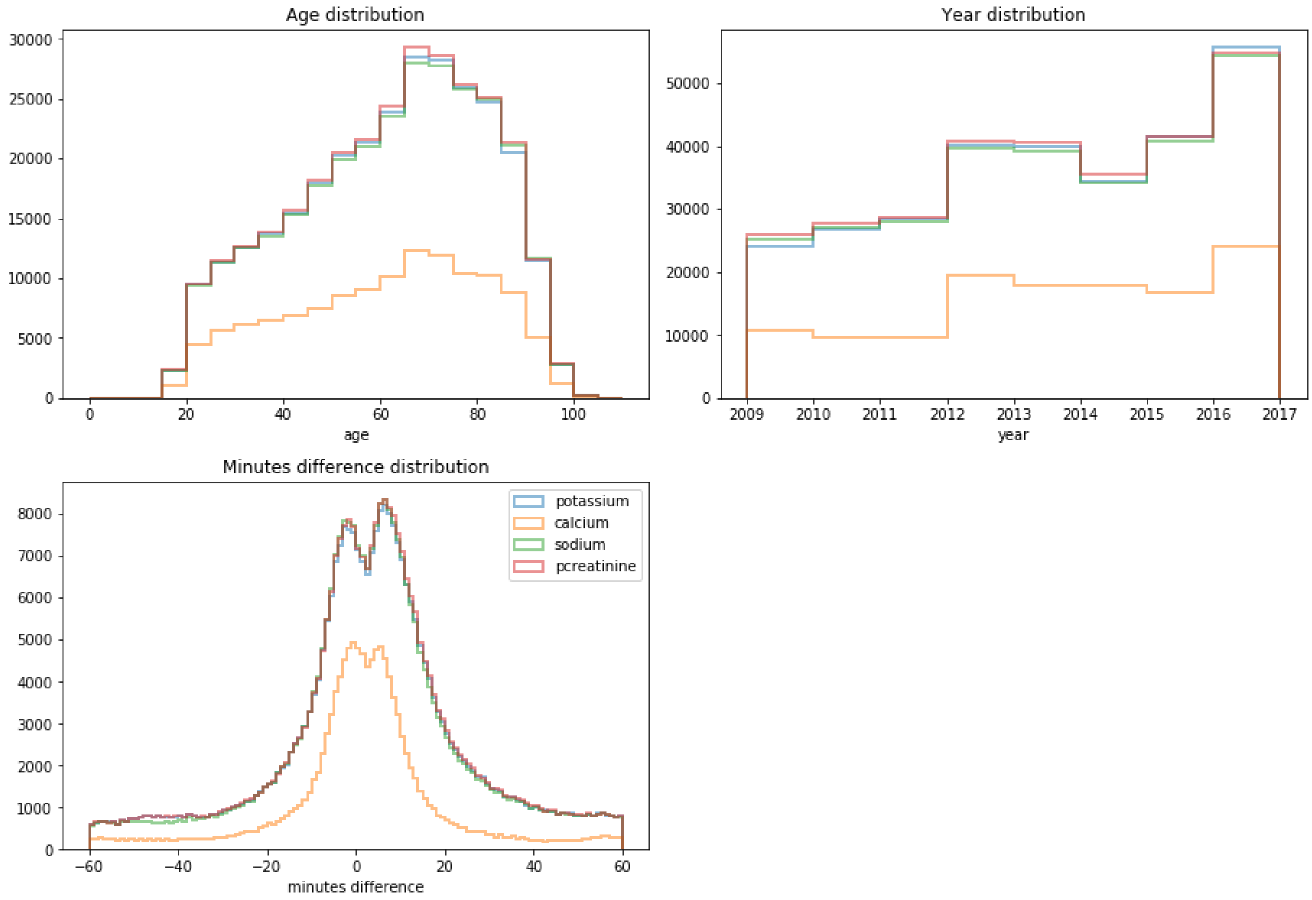

Figure S-1. Histogram of metadata age (top left), recording year (top right) and minutes difference between ECG recording and blood measurement (bottom) for our four datasets.

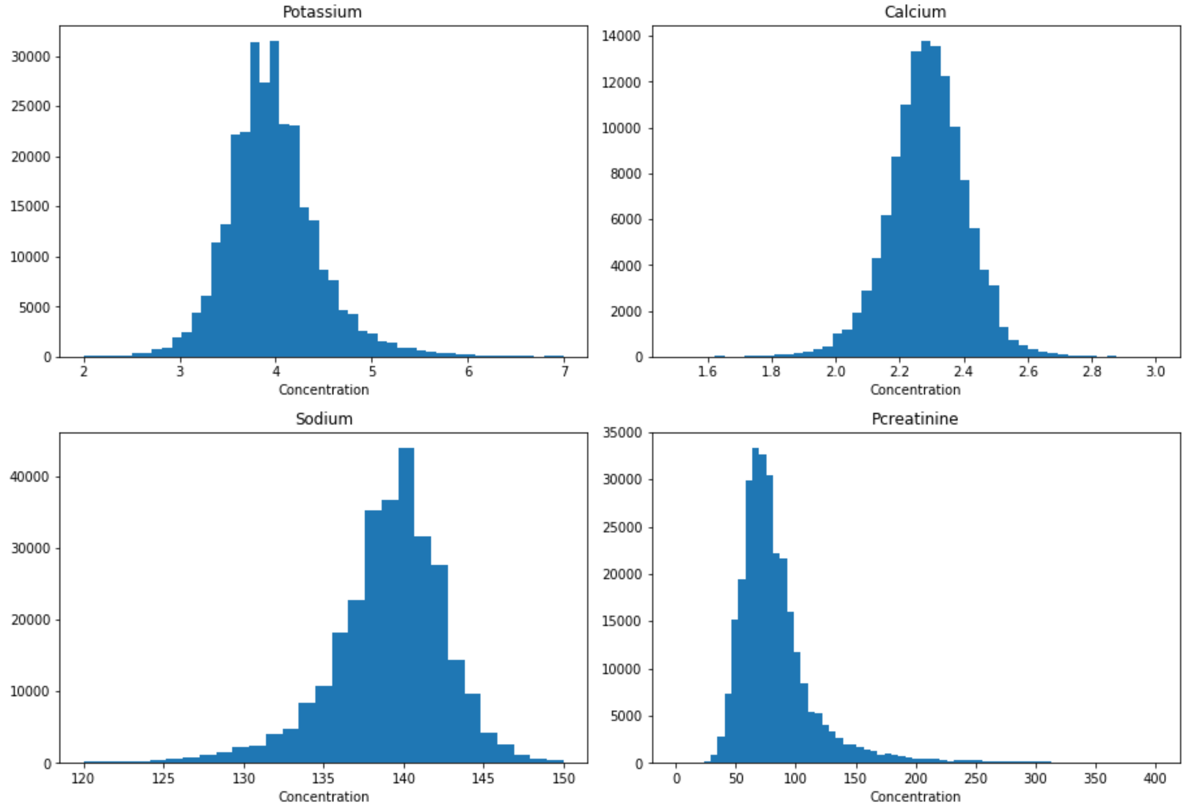

Figure S-2. Histogram of the electrolyte concentration values for our four datasets.

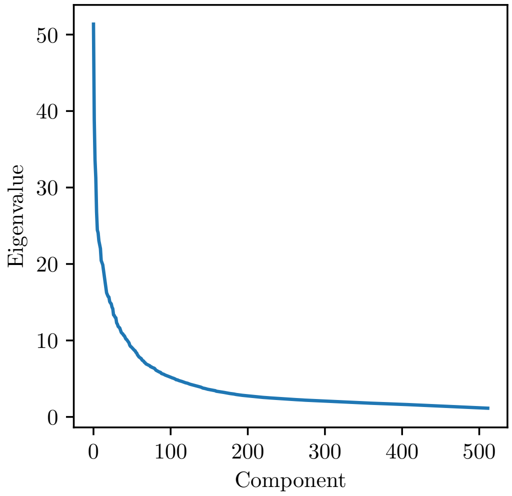

Figure S-3. Eigenvalues of PCA components fit on train set. We show the first 512 eigenvalues of possible $8 \cdot 4096 = 32\,768$ ones. We choose to reduce the dimensionality of our signal to 256 as it covers most information according to this figure.

B Training Details

B.1 Network Architecture

We use a modified ResNet which was first developed in Ribeiro et al. [10], and later also in Lima et al. [13], which also provides a public GitHub repository: `https://github.com/antonior92/ecg-age-prediction`. We adjust the last linear layer of the model for the different tasks, for example, different number of outputs for classification.

Our ResNet backbone from [10] consists of one convolutional layer followed by four residual blocks. The convolutional layer and each residual block modify the sequence length by {4096, 1024, 256, 64, 16} and the filter size by {64, 128, 196, 256, 320}. We use a kernel size of 17 and a dropout rate of 0.5.

B.2 Hyperparameters

We use the default training hyperparameters from the original network architecture repository. The only deviation is the number of epochs which we reduced from 70 to 30, since this is sufficient for our datasets to converge. The exact hyperparameters are listed in Table S-1.

Table S-1. Hyperparameters for training the DNNs

Hyperparameter	Value
optimizer	Adam
maximum epochs	30
batch size	32
initial learning rate	10^{-3}
learning rate scheduler	ReduceLROnPlateau
patience	7
min. learning rate	10^{-7}
learning rate factor	0.1

C Additional Results

Below we present additional results. First, we have a detailed performance table (more detailed than Table 3) for all electrolytes in Table S-2 for the random test set and in Table S-3 for the temporal test set. No significant difference in performance between the test sets is observed which shows that our model is robust to shift and trends over time.

Second, we list more results for classification and ordinal regression. In Figure S-4 we show the MAE for potassium and calcium which complements Figure 4 that shows the Macro ROC. Figure S-5 complements the electrolytes by showing the Macro ROC and MAE for the other electrolytes (creatinine and sodium).

Third, we show additional results for probabilistic regression. Figure S-6 gives the calibration plot for potassium. The tables in Table S-4 and Table S-5 contain numeric details about the sparsification plot for more uncertainties, and the correlation between MSE and the variance to quantify the uncertainty calibration. Table S-6 lists the results of the OOD experiments. While the experiments for the SNR are expected (larger MAE and uncertainties for lower SNR), the results for masking are not as clear. While the MAE still increases, notably, especially the epistemic ensemble uncertainty decreases. This means that there is less variance in the mean predictions between the different ensemble members. Finally, Figure S-7, Figure S-8 and Figure S-9 yield the results for the remaining electrolytes that were previously shown for potassium alone.

Table S-2. Regression performance on the random test dataset: Table shows metrics for different electrolytes of the regression models from "Results", "Deep Direct Regression". Target variance refers to the variance of the dataset and therefore yields a worst case MSE performance (since a model with that MSE just predicts the mean of the dataset).

Electrolyte	MSE (sd)	MAE (sd)	Target variance	normalized MSE (sd)
Potassium	0.1524 (0.0259)	0.2846 (0.0152)	0.2158	0.6013 (0.1021)
Calcium	0.0148 (0.0002)	0.0877 (0.0005)	0.0159	0.8625 (0.0088)
Sodium	12.5933 (0.1108)	2.5123 (0.0156)	13.1026	0.8445 (0.0074)
Creatinine	3719.8727 (86.0351)	26.6929 (1.118)	4074.4715	0.7097 (0.0164)

Table S-3. Regression performance on the temporal test dataset: Table shows metrics for different electrolytes of the regression models from "Results", "Deep Direct Regression". Target variance has same meaning as in Table S-2.

Electrolyte	MSE (sd)	MAE (sd)	Target variance	normalized MSE (sd)
Potassium	0.1319 (0.0171)	0.262 (0.0127)	0.1987	0.5203 (0.0675)
Calcium	0.0201 ($\leq$ 0.0001)	0.1059 (0.0003)	0.0167	1.1742 (0.0028)
Sodium	12.0118 (0.084)	2.3903 (0.0093)	12.7672	0.8055 (0.0056)
Creatinine	2973.3104 (156.24)	24.5017 (1.2979)	3370.132	0.5673 (0.0298)

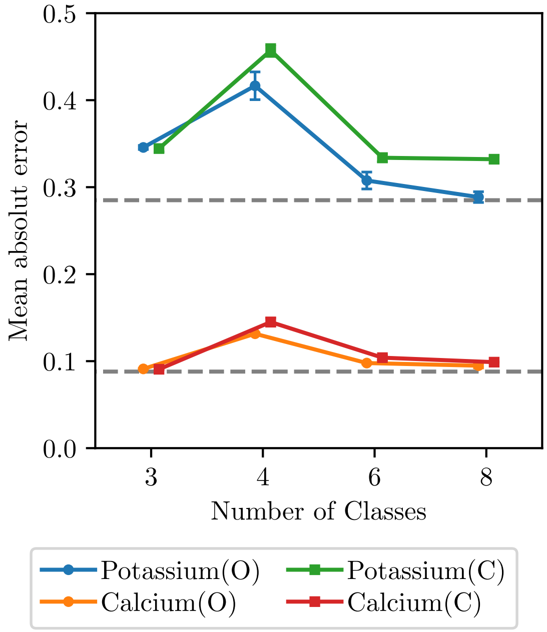

Figure S-4. Classification (C) and Ordinal regression (O) MAE: Similar to Figure 4, we plot the MAE against the number of classes. The dashed line is the MAE of the corresponding deep direct regression model.

Table S-4. Sparsification against MAE: Numbers in columns show different levels of sparsification (in per cent), and the corresponding row shows MAE values. This table gives the numeric values of the bottom right plot of Figure S-6.

	**25**	**50**	**75**	**100**
Aleatoric Gaussian	0.213(0.007)	0.228(0.008)	0.250(0.009)	0.283(0.009)
Epistemic ensemble	0.235(0.006)	0.246(0.009)	0.259(0.010)	0.283(0.009)
Epistemic Laplace	0.249(0.014)	0.260(0.021)	0.271(0.019)	0.283(0.009)
Aleatoric Gaussian + Epistemic ensemble	0.211(0.005)	0.227(0.007)	0.249(0.008)	0.283(0.009)
Aleatoric Gaussian + Epistemic Laplace	0.212(0.007)	0.228(0.008)	0.250(0.009)	0.283(0.009)
Epistemic ensemble of direct reg.	0.227(NA)	0.238(NA)	0.249(NA)	0.274(NA)

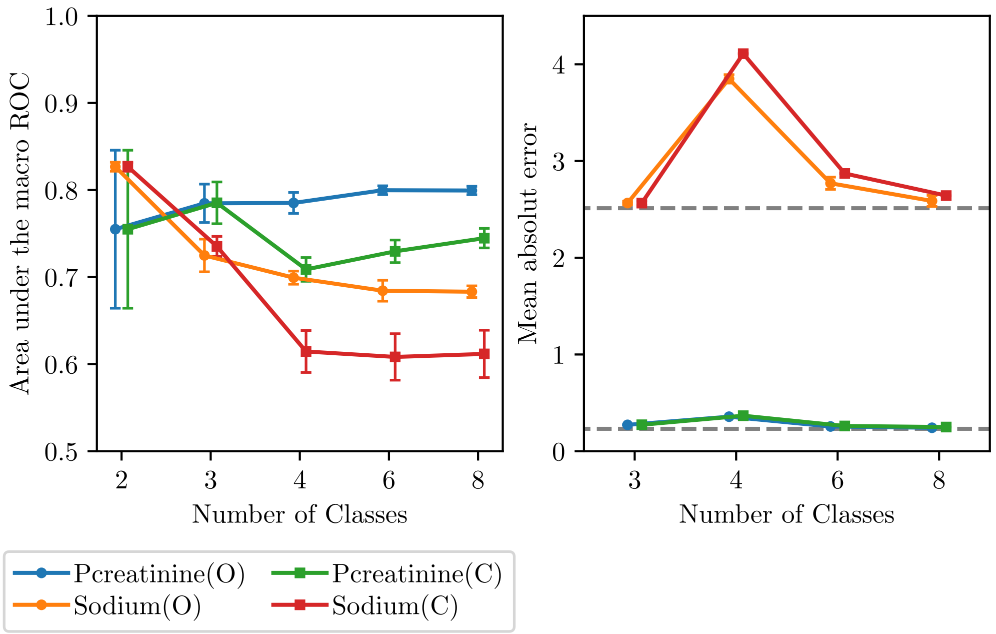

Figure S-5. Classification (C) and Ordinal (O) regression: Same plot as Figure 4 and Figure S-4 but for creatinine and sodium (here we only used 4 seeds for shown mean and sd).

Table S-5. Correlation between MSE and Variance: we correlate the MSE with the variance from different uncertainties. A correlation of 1 would indicate perfect calibration.

	Correlation
Aleatoric Gaussian	0.225(0.066)
Epistemic ensemble	0.218(0.010)
Epistemic Laplace	0.068(0.034)
Aleatoric Gaussian + Epistemic ensemble	0.255(0.039)
Aleatoric Gaussian + Epistemic Laplace	0.225(0.066)
Epistemic ensemble of direct reg.	0.225(N/A)

Table S-6. OOD experiments: This is an extended table from Table 5. SNR X refers to OOD experiments with varying SNR; Mask X refers to OOD experiments where X per cent of the data is masked.

	MAE	Aleatoric Gaussian	Epistemic ensemble	Epistemic Laplace	Epistemic direct reg.
Baseline	0.304(0.021)	0.389(0.012)	0.121(0.048)	0.022(0.003)	0.099
SNR 10	0.330(0.016)	0.399(0.012)	0.149(0.041)	0.028(0.009)	0.134
SNR 1	0.368(0.026)	0.480(0.078)	0.184(0.075)	0.049(0.031)	0.154
Mask 25	0.300(0.008)	0.386(0.015)	0.091(0.015)	0.022(0.001)	0.098
Mask 50	0.311(0.005)	0.388(0.008)	0.070(0.010)	0.020(0.002)	0.073
Mask 75	0.334(0.001)	0.385(0.004)	0.047(0.005)	0.018(0.002)	0.054

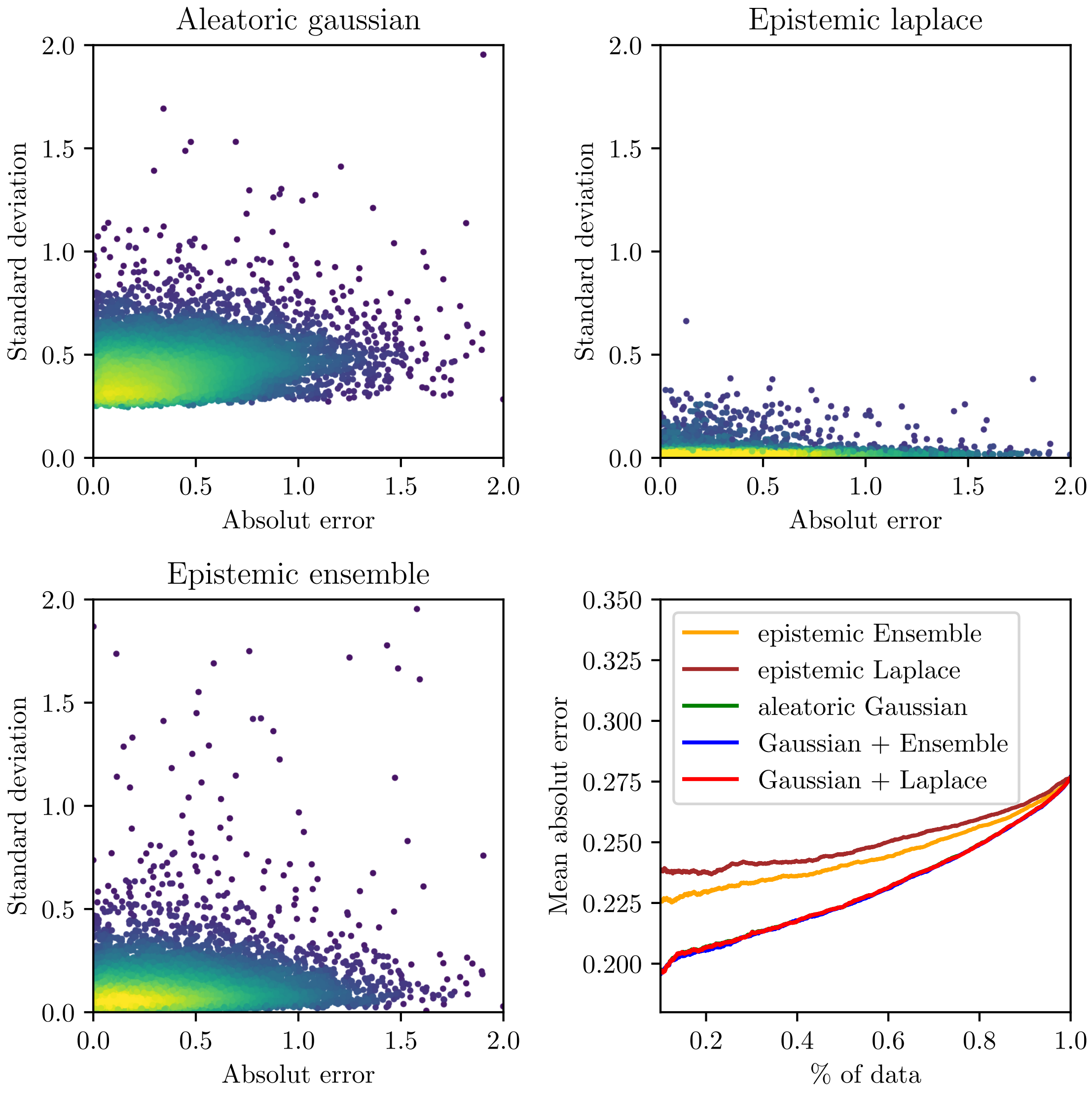

Figure S-6. Calibration plot, potassium: *Top row and bottom left:* calibration plots as standard deviation vs. absolute error (to have the same units) for different uncertainties. Colours indicate frequency by a fitted Gaussian kernel density estimate. A perfectly calibrated model would follow the diagonal. *Bottom right*: sparsification plot with more results than in the main paper.

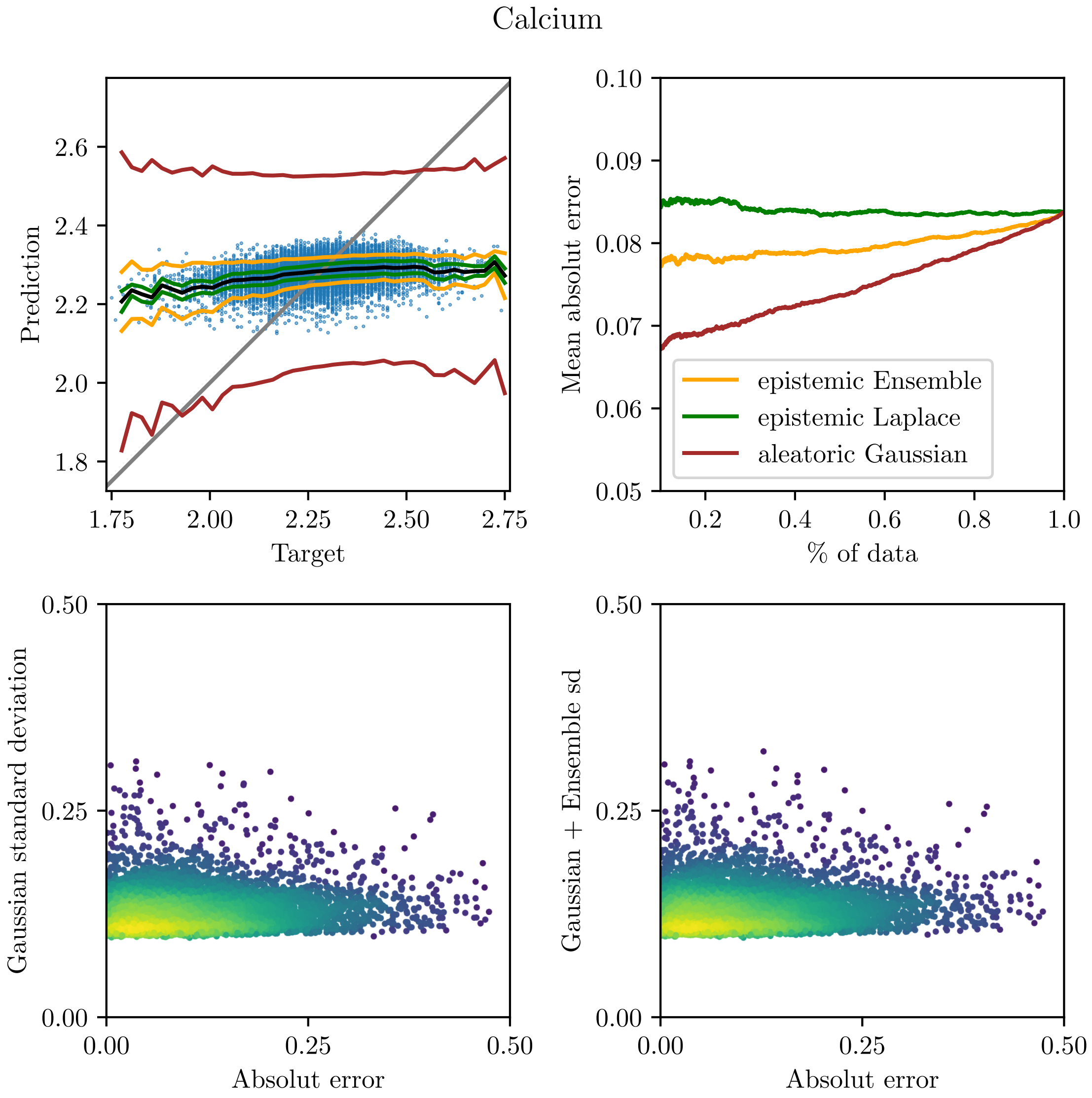

Figure S-7. *Top left*: prediction vs target plot including various uncertainties. *Top right*: sparsification plot. *Bottom*: Calibration plots between different uncertainties and absolute error. The frequency of samples is highlighted by colour which is fitted with a Gaussian kernel density estimate.

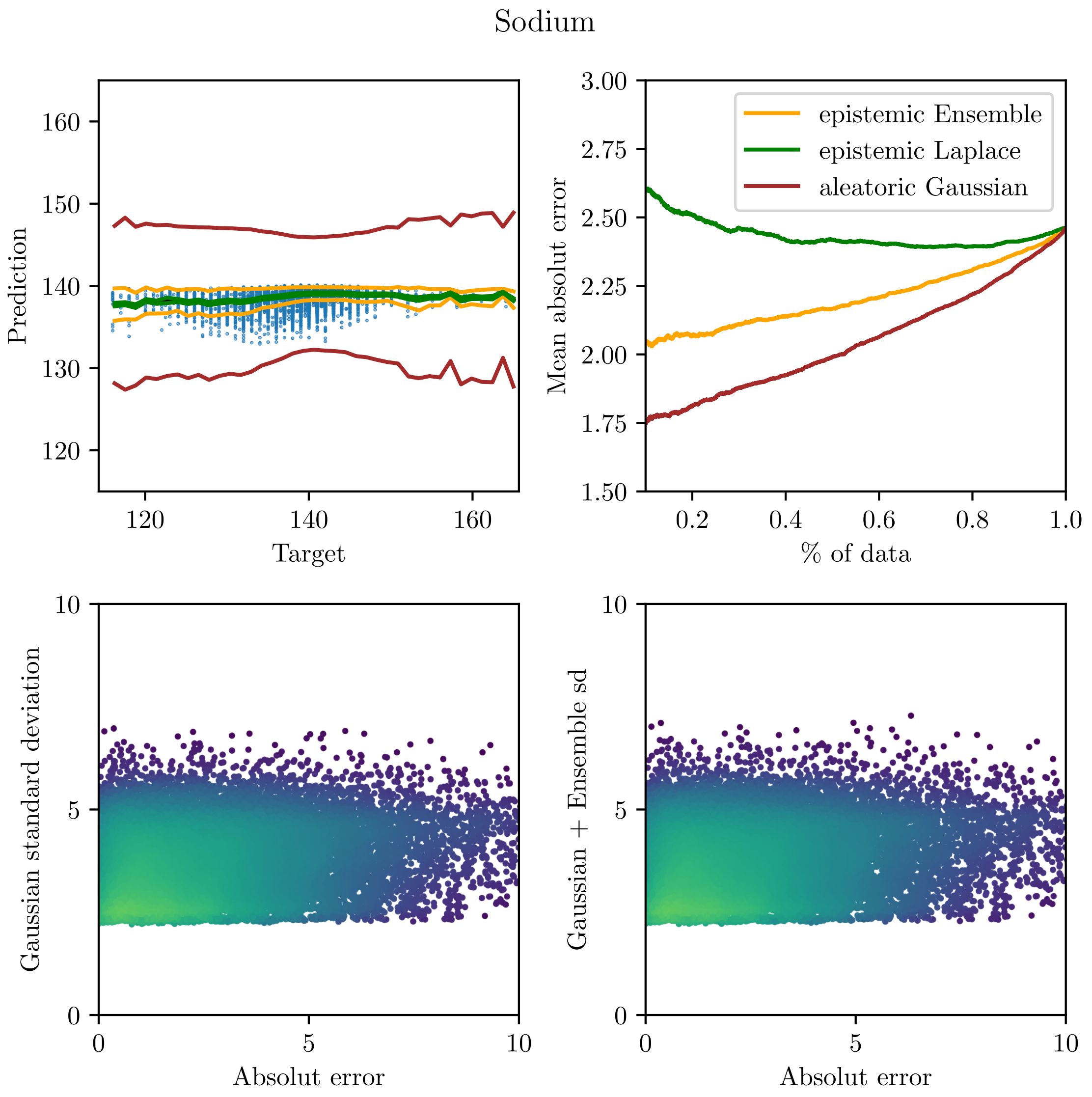

Figure S-8. Same results as Figure S-7 but for sodium.

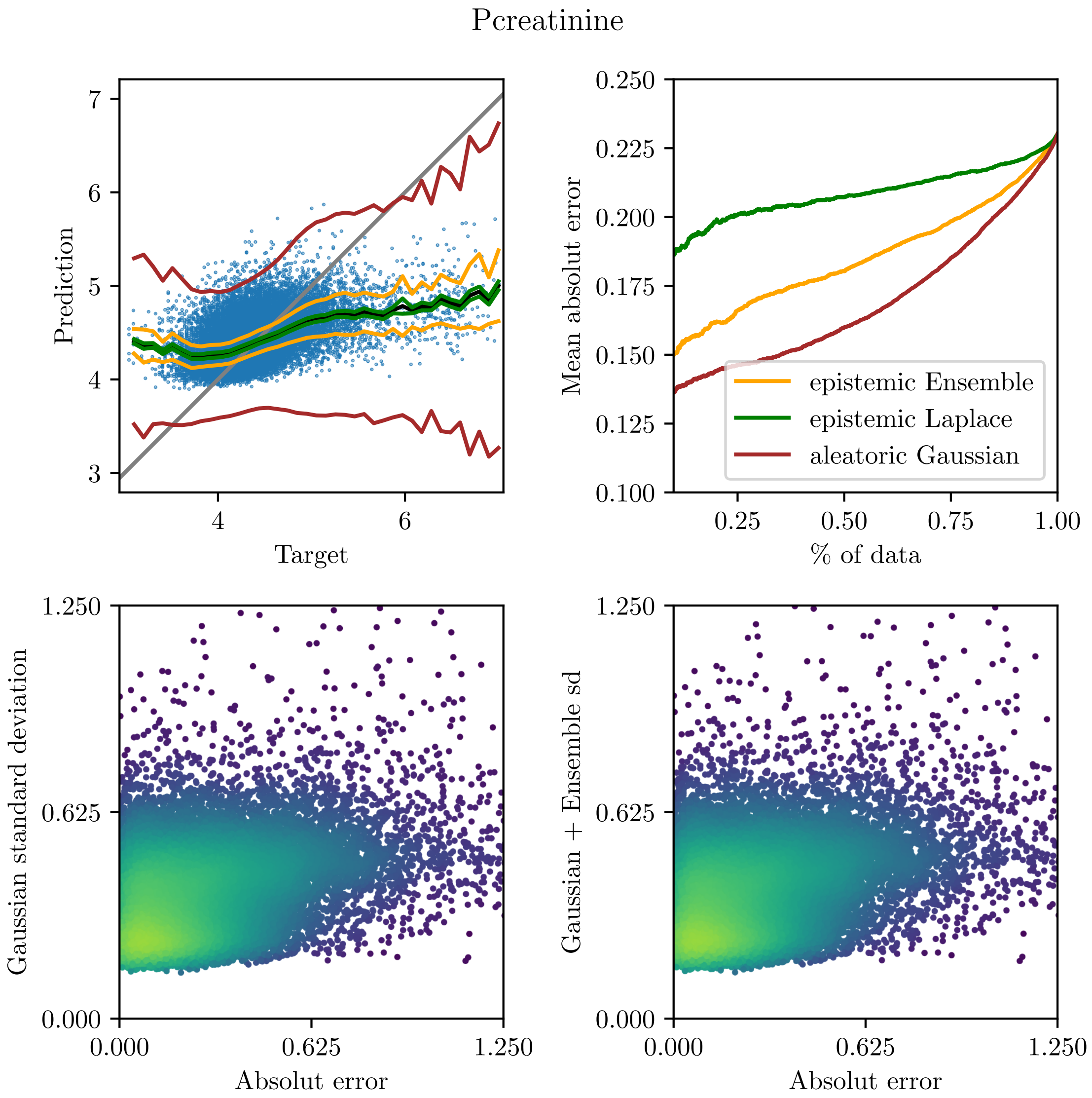

Figure S-9. Same results as Figure S-7 but for creatinine, in the log-transformed space due to the heavily skewed distribution of creatinine.
